# Dr. Granville's Essay on Egyptian Mummies

**Published:** 1826-07

**Authors:** 


*An Essay on Egyptian Mummies; with Observations on the Art of Embalming among the Ancient Egyptians.* By A. B. GRANVILLE, M.D. F.R.S. &c. &c. &c.

[Philosophical Transactions, 1825.]

DID the first idea of *Mummication* (we have not coined this word) or the art of preserving the frail memorials of a former existence, spring from vanity, self-love, or friendship? Or was it founded, as we are told, in "religious principles—the desire of preserving the mortal remains of the meritorious—the noble ambition of transmitting down to posterity the fabric of man himself in conjunction with the monuments of his own genius

—or a tribute to filial, parental, or conjugal affection?"* Whatever were the causes (and doubtless there were many) they appear to have operated in all ages and in all countries. In the old as well as in the new world—in the various islands scattered between both, we find *Mummies*, preserved with greater or less skill—and we have not to go beyond Westminster Abbey or Windsor, to find Mummies of yesterday, as well as of centuries gone. And what use can this preservation answer—what principle can it fulfil? Were the practice universal, this earth would become one vast catacomb—or covered with "cities of the dead." We consider the practice of embalming as an act most contrary to nature, to religion, and to reason. Contrary to Nature, because her laws universally act against, and ultimately frustrate every attempt to preserve from decomposition the material tenements of life:—contrary to Religion, which wisely consigns "dust to dust:'†'—contrary to Reason, which clearly dictates that the putrefying exuviæ of man and all living creatures should be resolved into their constituent elements, and mingled with earth, water, or air, as soon as possible after the departure of the vital spark. Upon what plea can the Christian wish to preserve this earthly tabernacle, after he has shuffled off the mortal coil? Not from the hope of a resurrection. The omnipotent fiat which shall clothe once more with flesh the wandering spirit, will need little aid from cerecloth, natron, or frankincense! But man still clings to the material emblems of life; and however unphilosophic, the *feeling* must be allowed to be natural and amiable, which cherishes the remains of those whom we loved or reverenced in this sublunary scene. Hence we find means taken in all countries and ages to arrest the destructive tooth of time

* De Lens on Mummies.

† It is difficult to say whether the idea of preserving the material fabric of man be in favour of or against the belief in the soul's immortality. It is said the Egyptians thought that the soul departed from the body, at the time of death, and continued in a separate state of existence for the space of 3000 years, when it again took possession of its original tenement, provided that tenement was preserved—otherwise it was obliged to pass into the body of some animal. This idea presupposes the soul's immortality, and would explain the great attention paid to the act of embalming, and the doctrine bears some remote analogy to the Christian doctrine of the resurrection. On the other hand, a belief in the soul's immortality and existence in another state, gives but little encouragement to the preservation of the clay which it once animated. There is no proof indeed of this doctrine among the Egyptians, and therefore embalming was probably founded on some mysterious dogma of their superstition, unconnected with belief in a future state of existence.

on the lifeless fabric of man.* No people on earth have been so successful in this contest as the Egyptians. Their pyramids and their mummies have outlived all record, and almost all conjecture of their own age or origin—and hence the curiosity of mankind respecting these monuments of antiquity has been, and still is, boundless. This curiosity (we now confine ourselves to the subject immediately under review) is not irrational or idle, inasmuch as we have in these mummies the skeletons of people who lived three thousand years before our own time—and consequently we have the opportunity of comparing them with races of men now in existence. This comparison it is true, has led to little importance of result. Another object of curiosity is, the method by which the Egyptians preserved their dead. Dr. Granville conceives that he has imitated the process. But the space of four years is a small speck on the stream of time, compared with three thousand.†

The Egyptian practice of embalming is alluded to in scripture—indeed, Joseph caused his father to be embalmed, and he describes the process as lasting forty days. Various accounts of the mummies have been published, and various specimens examined at different times and in different countries; but it has been subsequent to the French and English expeditions to that interesting land of antiquities, that the immense catacombs and "cities of the dead" have been thoroughly explored. Messrs. Bouyer, Jomard, and Larrey have published most interesting memoirs on the subject, in the great work on Egypt not yet finished in Paris. Baron Larrey, in particular, has made many important remarks on the *races* of the people now presented to us as mummies. From an examination of the crania, the Baron appears to have proved, what historians had conjectured, that the Egyptians have really descended from the people of Abyssinia and Ethiopia. The Copts are allowed by all to be descended from these last people, and their skulls correspond precisely with those of the ancient mummies.

Those who are desirous of perusing a very minute account of the various appearances, as well as the ways in which mum-

* The modern practice of covering our walls with the portraits of our forefathers and friends is a decided improvement on the mummy furniture of the Egyptians and Peruvians. In contemplating the *former*, there is a mixture of pleasure with melancholy, resulting from the *presence* of our friend or parent's image—while the sight of the shrivelled corpse inspires us only with horror of death.

† A great deal of information is obtainable from Mummies respecting the manners, customs, arts, and manufactures of the countries where the mummies are preserved.

mies are preserved, may consult the Memoirs above alluded to, or a very copious account of these Memoirs in the 11th and 34th volumes of the *Dict. des Sciences Medicales,* under the heads "*Embaumement,*" by Pelletan, junior, and Momie, by De Lens.

We shall now proceed to notice some of the particulars observed by Dr. Granville, in his dissection of the mummy which came into his possession, and which we had an opportunity of seeing, while the doctor was examining its interior. It appears that few of the mummies, hitherto examined in this country, have been good specimens—many of them, indeed, have been complete impostors—as was the case in two instances, where Blumenback found, in one, only a bundle of bandages—and, in the other, was the mummy of an Ibis! It is curious that even the French travellers and literati before alluded to, should have met with few, if any, mummies having any of the viscera preserved—at least, they make very little mention of such appearances. They all represent the cavities of the cranium, thorax and abdomen, as filled with bituminous substances, and seem to think that the viscera were either extracted or destroyed by the injection of corroding substances.

On opening the abdomen, "the objects which then presented themselves were a portion of the stomach adhering to the diaphragm, the spleen much reduced in size and flattened, attached to the super-renal capsule of the left kidney, and the left kidney itself, imbedded in, but not adhering to, the latter, and retaining its ureter, which descended into the bladder. This, as well as the uterus and its appendages, were observed in situ, exhibiting strong marks of having been in a diseased state for some time previously to the death of the individual. Fragments only of the intestinal tube could be found, some of them of considerable dimensions, and among them part of the cæcum, with its vermiform appendix, and portions of the ileum. Several large pieces of the peritoneal membrane were likewise observed." 28.

There were also found several masses of a brittle resin, and some pieces of myrrh, which seemed to have been forced up to fill the cavity of the abdomen, after the removal of the larger portions of intestine and their contents through the anus—for in this mummy there was no lateral incision. No traces of the right kidney or of the liver could be found. Among the debris of the abdominal viscera, the late Dr. Baillie detected the gall-bladder slightly lacerated, but in other respects perfect, with some parts of its ducts attached. In the thorax the heart was found *in situ,* suspended by its own blood-vessels, and attached to the lungs, which were in a state of considerable preservation. In the cranium, no part of the brain, nor of its investing membranes, was to be seen. The *former* must have been extracted through the nostrils, as the œthmoid bone was perforated—the membranes were probably destroyed by some hot or corroding substance injected through the same opening. The eyes had not been disturbed—the *tongue* of this Egyptian dame was still in excellent preservation, as were the teeth. Dr. Granville conjectures the age of this female to have been about 50 or 55, and that she died of ovarian dropsy—hence this is probably the oldest pathological preparation in the cabinets of the curious.

Dr. Granville has made a great many curious observations and experiments on the substances employed in embalming this mummy, and on the mode in which they were used. These we find it difficult to abridge, and have not room for the whole. He has also imitated the process which he supposes to have been employed by the Egyptians, and with complete success, as far as a few years may be considered a trial.* The main steps of this process are supposed to be these. *First*, The evisceration (in the great majority of cases) of the different cavities.—*Secondly*, Covering the body with quicklime for a few hours, and then rubbing off the cuticle with some blunt instrument.—*Thirdly*, Immersion of the body in a vessel containing a liquefied mixture of wax and resin, with, perhaps, some unessential bituminous substance, in which the *patient* was boiled for a certain number of days, so that the liquefied mixture might penetrate the minutest structure of the body.—*Fourthly*, The tanning process, by means of some vegetable astringent and saline substance, the precise nature of which cannot now be discovered—and *lastly*, The bandaging, in which act the Egyptians must have been exceedingly expert—much more so, indeed, than our best modern surgeons.

Such were the means, or supposed means, which vain and superstitious man put in force to prolong the material existence of this inanimate envelope, after having exhausted every resource of art to ward off the stroke of inexorable DEATH ! "*Qui*

* "There were exhibited after the meeting four different specimens of imitative mummies, each of them illustrative of one or two of the successive stages of the process of embalming detailed in this Essay; the last being intended to illustrate all the stages together, and exhibiting a close resemblance to the Egyptian mummy itself. A still-born child had been employed for the purpose, and this modern mummy has now been in existence upwards of three years, without bandage or covering of any kind, exposed to all sorts of temperature and rough usage without betraying the slightest vestige of decay or putrefaction. It is rather darker than the Egyptian mummy from the circumstance of a too concentrated solution of tannin having been employed in preparing it." 47.

*mortem evitare non possunt, corporis saltem gaudeant duratione.*" By these means ancient Memphis, peopled the plains of Saggarrah, with human monuments of antiquity more durable than the marble or the granite of its mouldering and now entombed ruins.—By these means the hundred-gated Thebes vomited through her lofty portals those myriads of her children who were to crowd the "cities of the dead," in the heart of the neighbouring mountains, for 90 generations. There they awaited, during this long and dreary period, the advent of the wandering spirit, promised or predicted by priest or prophet, that was once more to animate their shrivelled frames, and lead them forth to behold the cheerful light of heaven. It did come—but it was the *spirit of curiosity,* in human shape, from the four corners of the earth. These sanctuaries were at length profaned.—The mighty lords and lovely women of Egypt—gods and men—apis and osiris—the ibis and the ichneumen—were all unhoused and doomed to the same indignities! The body of Sesostris himself was probably sold on the banks of the Nile for a couple of dollars. His queen or his daughter may have been the identical mummy, whose heart was torn from her breast by the rude hand of Dr. Granville!* A queen or a princess of Egypt may thus have been unswathed and exposed naked to the eye of the antiquarian, and her bones and bowels *exhibited* at Somerset House! To crown all, some blighted abortion of Brownlow-street, has been salted, tanned, and smoaked to rival the wife and the daughter of a Sesostris, a Psamureticus, or a Ptolemy! This picture of the vanity of all human hopes and wishes—as well as the instability of all sublunary things, conveys a moral lesson as valuable as it is obvious. The impression which it is calculated to leave on the mind we will not run the risk of weakening by further reflexions.

---

* "When I beheld before me the heart of an Egyptian female whom imagination, aided by historical records, may fancy to have been cotemporary with the great Sesostris, I could not help feeling a degree of enthusiasm, a portion of which, me thought, I could impart to others."—*Granville, p.* 49.

